# Development of 3D Wafer Level Hermetic Packaging with Through Glass Vias (TGVs) and Transient Liquid Phase Bonding Technology for RF Filter

**DOI:** 10.3390/s22062114

**Published:** 2022-03-09

**Authors:** Zuohuan Chen, Daquan Yu, Yi Zhong

**Affiliations:** School of Electronic Science and Engineering, Xiamen University, Xiamen 361005, China; xmuchenzh@stu.xmu.edu.cn (Z.C.); zhongyi@xmu.edu.cn (Y.Z.)

**Keywords:** RF filter, wafer-level TSV/TGV capping packaging, transient liquid phase bonding

## Abstract

The development of 5G mobile communication created the need for high-frequency communication systems, which require vast quantities of radio frequency (RF) filters with a high-quality factor (Q) and low inband losses. In this study, the packaging of an RF filter with a through-glass via (TGV) interposer was designed and fabricated using a three-dimensional wafer-level package (3D WLP). TGV fabrication is a high-yielding process, which can produce high precision vias without masking and lithography and reduce the manufacturing cost compared with the through silicon via (TSV) solution. The glass interposer capping wafer contains Cu-filled TGV, a metal redistribution layer (RDL), and the bonding layer. The RF filter substrate with Au bump is bonded to the capping wafer based on Au-Sn transient liquid phase (TLP) bonding at 280 °C with a 40 kN (approximately 6.5 MPa) bonding force. Experimental results show that shear strengths of approx. 54.5 MPa can be obtained, higher than the standard requirement (~6 MPa). In addition, a comparison of the electrical performance of the RF filter package after the pre-conditional level three (Pre-Con L3) and unbiased highly accelerated stress (uHAST) tests showed no difference in insertion attenuation across the passband (<0.2 dB, standard value: <1 dB). The final packages passed the reliability tests in the field of consumer electronics. The proposed RF filter WLP achieves high performance, low cost, and superior reliability.

## 1. Introduction

As more components are incorporated into radio frequency (RF) front-end modules, devices are more densely packed, and more heat is produced in the modules. The integration of RF filters in the front-end module demands further miniaturization and higher temperature stability [1].

RF filter demands excellent frequency band selectivity, high quality, and low insertion loss. Surface acoustic wave (SAW) and bulk acoustic wave (BAW) filters become the essential technique route. SAW filter combines low insertion loss with good suppression performance. However, it typically works below 1.5 GHz-band and is easily affected by temperature [2]. BAW filters are favored in many high-frequency applications due to their excellent frequency band selectivity, especially in 4G and 5G communication terminals. A BAW filter is usually packaged using thin-film cavity acoustic resonator (FBAR) technology. Its typical structure is two metal electrodes clamped to the piezoelectric film [3]. The piezoelectric film is sensitive to any additional mass loading on the surface, such as humidity or any corrosion. Thus, efforts in packaging are increasingly important. For propagation of the bulk wave, the package requires a cavity structure above the chip surface to prevent moisture corrosion and provide a stable/safe environment [4]. In addition, the parasitic effects of packaging for RF or microelectromechanical system (MEMS) devices should be minimal.

WLP with low insertion loss offers a cost-effective and promising solution for the RF filter packaging and 3D integration [5,6,7,8]. There have been several efforts to package these high-Q FBAR or RF MEMS devices using wafer capping bonding and 3D through vias vertical feedthrough technologies [7,9,10,11]. Wafer bonding methods include anodic and direct (or fusion) bonding [12,13,14]. Direct bonding provides reliable, low-temperature bonding solutions by adding polymer adhesive metallic material to the bonding interface. Metallic bonding is a popular method of hermetic packaging, as it provides mechanical stability and can be integrated into various fabrication processes. Meanwhile, metallic bonding can be achieved as a preferred solution through various means, such as thermocompression bonding, eutectic bonding, and TLP bonding [15]. TLP bonding provides a low bonding temperature and a higher remelting temperature, beneficial for RF or MEMS device packaging. Some research groups have demonstrated TLP wafer-level bonding using Cu-Sn, Au-Sn, and Cu-Sn-In material systems [9,16,17,18]. The Au-Sn material system provides low-cost TLP bonding, widely applied in electronic encapsulation. Consequently, some combination of pressure, temperature, bonding layer thickness, Au thickness, and a narrow bonding frame for RF devices are required.

Generally, the following criteria are used to realize packages compatible with RF or MEMS devices [19]: (1) Ensure good consistency in the electronic characteristics before and after packaging (inaccurate delta value is less than 1 dB in peak insertion loss (IL) of the passband for RF filter package); (2) Interconnection construction should be able to realize dense packaging of low-loss RF circuits; (3) The package must ensure the reliable operation of the device; (4) The packaging processes must be compatible with various MEMS and complementary metal-oxide-semiconductor (CMOS) technologies on a wafer.

How to build up robust and cost-effective encapsulation becomes a critical issue. For filter packaging, a wafer-level bonding process with TSV is commonly used for the high-Q FBAR resonators. For example, M. Small et al. [7]. have presented an Avago microcap process. The FBAR device was fabricated on one silicon wafer while a second “cap” wafer contained TSVs, Au pads, a seal ring structure, and a recessed air cavity. The lid wafer was Au- thermocompression bonded to a base FBAR wafer to make a robust, hermetic package. D. Xu et al. [20] presented a wafer-level vacuum package for a micromachined thermoelectric infrared sensor based on Au-Au thermocompression bonding technology. H.R. Tofteberg et al. [21] presented a hermetic wafer-level Au-Au bonding in 350~450 °C temperature range. Bond strengths measured by pull tests ranged from 8 to 102 MPa. Al Farisi M S et al. [22] demonstrated a thermocompression bonding process utilizing an electroplated-planarized Au micro-sealing frame. The bonding temperatures of Au thermocompression bonding were lowered to 300 °C using electroplated Au with 10 μm height on a 4 inch Si wafer. And the 400 μm-thick capping layer of the SOI wafer is used as the capping layer. It is not advantageous for low cost, ultra-thin (package size < 300 μm, capping layer < 100 μm) hermetic packaging solution. Yamamoto S [23] designed a low-temperature hermetic packaging for microsystems using Au–Au surface-activated bonding. In this study, thin Au sealing rings (300–500 nm thick and 100 μm wide) were used as bonding layers. The technology is not achievable for a narrow bonding frame (here 100 μm).

B. Chen et al. [19]. have developed the fabrication of RF MEMS, which included TSV etching, void-free TSV plating, Cu-Sn diffusion-bonded to the device wafer, and RDL post-process on cap wafer. S.R. Gilbert et al. [6] have presented a wafer-level bonding packaging solution using a silicon lid wafer. Its main function is to provide TSVs connecting from the FBAR filter to the outside copper pads. These solutions reduced package profile and interconnect length and, in part, enhanced electrical performance over a chip area. However, during the TSVs fabrication process, this is much more complex. For example, high-speed etching has been combined with electrical isolation and diffusion barriers.

Hermetic materials include glasses, silicon nitride, and metals. In the WLP, many kinds of material, such as epoxies and organic polymers, are involved, which influence packaging lifetime. Glass wafers can be made out of different materials and processing capabilities to meet specific requirements [24,25,26]. Meanwhile, TGVs fabrication is a high-yielding process based on laser-induced deep etching (LIDE) technology. It can produce high-precision vias without masking and lithography and reduce the manufacturing cost compared with the TSV fabrication. And glass material is very attractive because it exhibits excellent hermetic performance and doesn’t cause outgassing compared to film lamination packaging. The glass is transparent, and we can track the packaging yield in real-time during processing. It is not advantageous for tracking the packaging yield for wafer-level packaging of RF MEMS devices by transfer bonding of silicon caps, especially after the bonding process. Therefore, it is commonly used in MEMS or RF field WLP. Yang et al. [27] presented a MEMS fabrication process with TGVs by laser drilling technology, and reliability concerns were overcome during the whole packaging process. Lee et al. [28] presented a wafer-level RF MEMS packaging structure with Cu-filled TGVs, and mechanical reliability was confirmed through a thermal shock test. However, studies on the 3D WLP with TGVs vertical feedback and cavity hermetic for RF filter were rarely reported in the available literature. Therefore, we are working on developing a 3D WLP for RF filter with a low cost, high performance, and superior reliability.

In this paper, we present a novel process development of wafer-level hermetic pack-aging with TGVs structure for RF filters, especially FBAR devices, which allows the co-integration of on-chip passive devices. To seal the internal structure, the packaging uses a TLP bonding with Au-Sn solder. A closed square loop of the Au-Sn bumping ring is fabricated at the chip’s edge area to make a cavity. The device’s pads inside the ring are used to support the weight of the back end of the line (BEOL) and the I/O interconnection. In Section 2, we propose the RF filter WLP structure and optimize the bonding structure. Section 3 introduces the TGV fabrication and bonding process for RF filter 3D integration. Section 4 discusses reliability evaluation strategies and the final packaging of RF filters using 3D/TGV technologies. Lastly, we conclude with some important findings and remarks in Section 5.

## 2. RF Filter the Package Design

### 2.1. 3D WLP Structure for RF Filter

**Figure 1:**
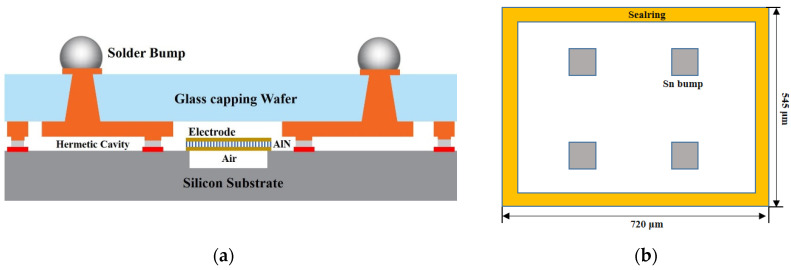
(**a**) Schematic structure of AlN RF filter WLP. (**b**) Top view of the capping wafer.

Figure 1a shows the schematic of the proposed AlN RF filter packaging structure. The device component, which has a size of 720 μm × 545 μm with a thickness of 300 μm (with ball), is composed of a two-part glass cap and device wafer. They are bonded through a closed square loop of Au-Sn soldering ring at the chip’s edge area using a wafer bonder (EVG 520). Full filling of the TGVs and metal traces were formed on both sides of the glass. Meanwhile, in Figure 1b, four metal pads inside the seal ring are used for I/O interconnection between the device and external signal source via the TGVs and RDL. The width of the Au and Sn layer is set to 43/27 μm, respectively. The cap wafer is an 8-inch glass wafer with a thickness of 100 μm. It has an internal cavity array with a depth of 20 μm to provide clearance for the device to work properly. TGV is fabricated by LIDE and filled by copper electroplating for electrical through. We retained the Au-Sn wafer bond proven to provide reduced size and a robust hermetic seal.

### 2.2. Optimization of the Bonding Structure and Bonding Parameters

#### 2.2.1. Optimization of the Thickness of the Sn and Ni Layer

**Figure 2:**
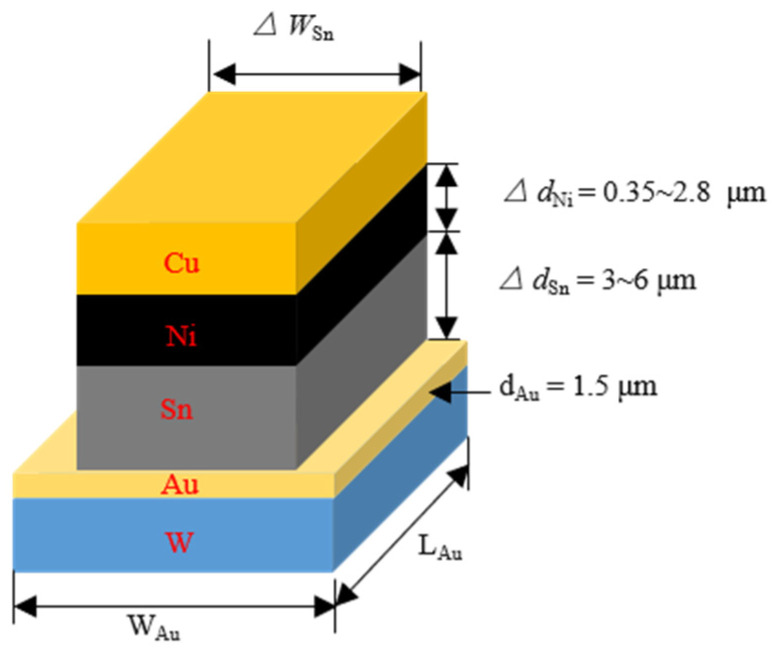
The simplified Au/Sn/Ni/Cu micro-joint.

As the bonding process is fluxless, systematic experimental work has been carried out to study the effect of thickness of the Sn layer in the seal ring and bump metallization on the reliability of the RF filter package. Firstly, too thick seal ring closed square will result in molten Sn overflow from the edge into the die area, which will result in a short circuit. Conversely, a too-thin Sn layer will cause poor wetting, affecting the bonding strength. Figure 2 shows the cuboid micro-joint, the effective Au concentration, set at 1.5 μm, and all Au atoms are dissolved in the solder, which can be calculated by Equation (1) [29].

(1) $$\begin{array}{l} C_{Au}\left(wt.\%\right)=100\frac{d_{Au}\rho_{Au}}{d_{Au}\rho_{Au}+d_{Sn}\rho_{Sn}}\\ \rho_{Sn}=7.3\;g/cm^{3}\\ \rho_{Au}=19.3\;g/cm^{3} \end{array}$$

Here, $C_{Au}$ is the effective Au concentration. $d_{Sn}$ and $d_{Au}$ are the thickness of the Sn and Au, respectively. $\rho_{Au}$ and $\rho_{Sn}$ are the densities of the Au and Sn, respectively. The critical thickness of the Sn layer is set to 3 μm, 5 μm, 6 μm. Applying Equation (1), the effective Au concentration $C_{Au}$:

(2) $$C_{Au}\left(wt.\%\right)=\{\begin{array}{cc} 56.93\% & d_{Sn}=3\;\mu m\\ 44.23\% & d_{Sn}=5\;\mu m\\ 39.79\% & d_{Sn}=6\;\mu m \end{array}$$

To control the diffusion process, a thin Ni-buffer layer was introduced into the bonding structure. The Ni layer has two main effects. First, it can prevent fast diffusion between the low-temperature Sn and Cu components during storage and the step of heating up during the bonding process. Second, the thin buffer layer dissolves into the Sn at the beginning of the soldering reaction. Then the diffusion between the solder materials and the Cu started, and finally, all solder was converted into intermetallic compounds (IMCs). As for improving reliability, control of Ni layer thickness becomes very important. The thickness of the Ni layer varied from 0.35~2.8 μm, while the Au layer was set at 1.5 μm to simulate the interfacial reactions during solid-state aging. We found that all the layers showed a decreasing trend in the shear strength during the early stage. Meanwhile, for the thinner Ni layer, the shear strength decreased to a lesser extent [30]. Based on the actual packaging process, the actual Sn layer thickness was set to 5 μm.

#### 2.2.2. Experimental Examination of TLP Bonding

**Figure 3:**
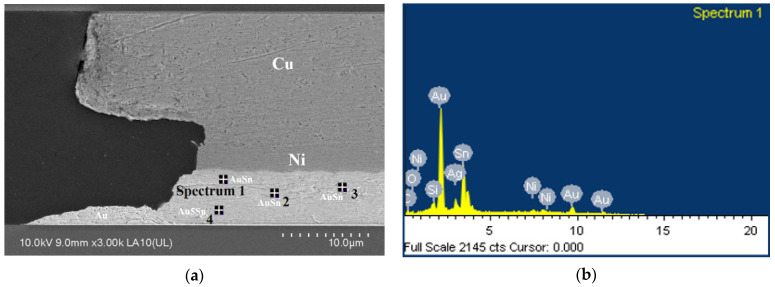
(**a**) the cross-sectional image of Au/Sn/Ni/Cu micro joint. (**b**) Au effective concentration.

Figure 3 shows the cross-sectional image of Au/Sn/Ni/Cu micro joint with 5 μm Sn layer after the bonding process. It can be seen that Au is consumed and formed IMCs. The Au effective concentration is 44.23 wt.% in the seal ring, and bumps if complete mixing is assumed.

In Table 1, energy-dispersive X-ray spectroscopy (EDX) analysis shows that the effective Au concentration is about 42 wt.% to 62.3 wt.%. According to the AuSn binary phase diagram [30], the Sn layer on the capping wafer is almost consumed, and the final microstructure should consist of Au5Sn (*ς*′ phase, as shown in spectrum 4) + AuSn (δ phase, as shown in spectrum 1) + AuSn2 (ε phase, as shown in spectrum 2, 3). There is no void at the interface between the Ni layer and the IMC layer. Meanwhile, Ag is detected in EDX shown in Table 1 (see spectrum 1 and spectrum 2), the main reason being that the electroplating liquid contains “Ag” impurity.

## 3. Packaging Process

**Figure 4:**
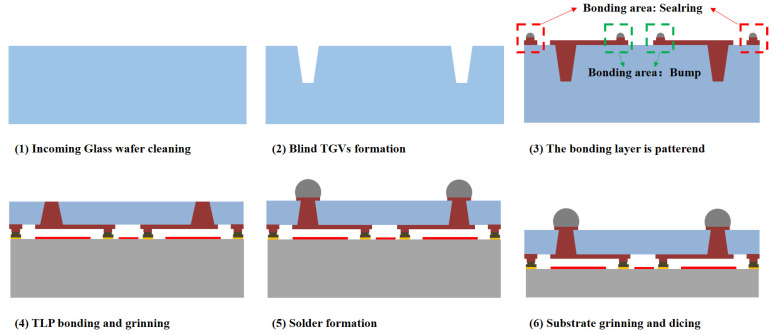
The process flow of the 3D WLP package.

The fabrication processes of the test vehicle for RF filter packaging involve a TGV cap wafer which includes transmission lines and RF filter fabrication process on the silicon substrate. The characteristic of RF filter WLP is determined by an accurate delta value that should not be greater than 1 dB in peak insertion loss (IL) of the passband. Figure 4 illustrates the process flow of the present 3D WLP for the RF filter. In this process flow, key processes include TGV cap wafer formation with transmission lines and through vias interconnection, Au-Sn TLP bonding, under bump metallization (UBM) formation, and solder ball.

### 3.1. Dummy Device Wafer Fabrication Process

**Figure 5:**
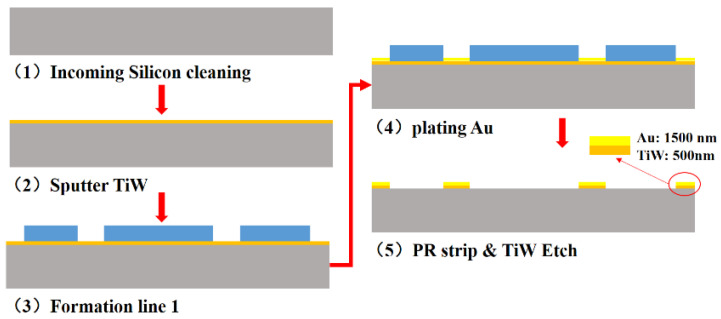
Dummy device wafer fabrication process flow.

**Figure 6:**
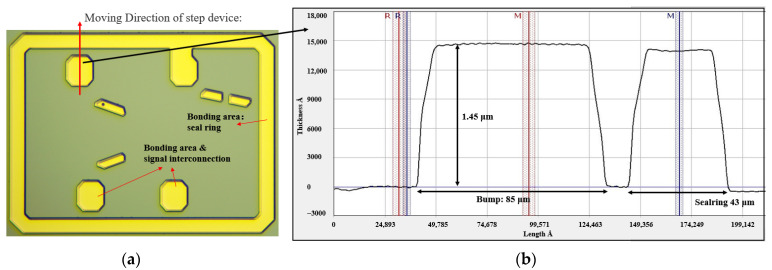
The structure of dummy device wafer; (**a**) The pattern of dummy device wafer; (**b**) The thickness and opening of Au bonding layer.

Figure 5 shows the fabricating process flow of the dummy wafer. Ti/W is sputtered on the silicon substrate as adhesive & seed layer. An organic photoresist 6μm thick is spin-coated and patterned. Figure 6a,b show that the pattern of the dummy device wafer and the opening of the seal ring and bump are 43.5 μm and 85 μm, respectively. In Figure 5 (4,5), 1.5 μm Au is electroplated as the bonding layer. Finally, the photoresist is removed, and the seed layer can be etched by the wet process.

### 3.2. Glass Cap Wafer Fabrication Process

The fabrication process of TSVs is comparatively complex. Its main fabrication processes are listed below.

- Step 1. SiO_2_ layer by PECVD.
- Step 2. Apply photoresist and mask, then use photolithography techniques to open vias on the SiO_2_;
- Step 3. RIE of SiO_2_;
- Step 4. Strip off the photoresist.

**Figure 7:**
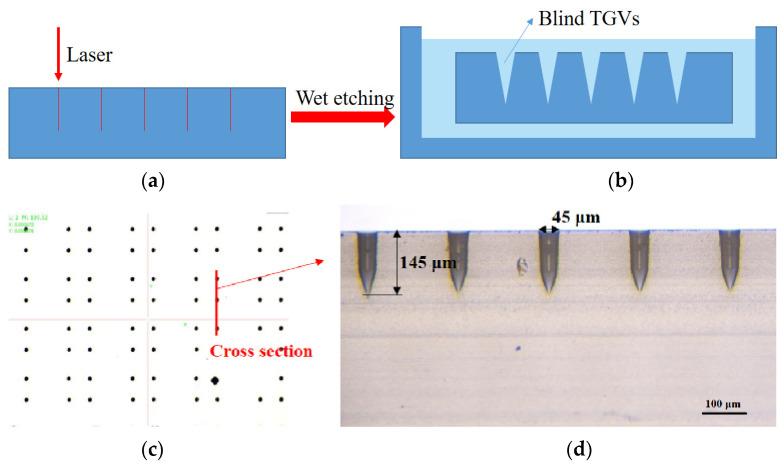
Optical micrographs (OM) of LIDE fabricated TGVs. (**a**) Schematic drawing of la-ser-induced. (**b**) The cross-section view of typical TGVs structure. The OM shows (**c**) the top view of a high-density array of TGVs, and (**d**) shows the cross-section of TGVs with aspect radio of 1:3.

To lower costs, LIDE is a high-yield process. It can produce high-precision vias without using masking and lithography and reduce the manufacturing cost compared with blind TSV fabrication. Meanwhile, LIDE technology can be used for different kinds of glass, such as quartz and borofloat. By selecting laser pulses, the concentration of HF solution and the chemical composition, fabricating TGV with the different profile can be achieved. In this paper, the key technology for fabricating a glass capping wafer includes blind TGVs formation, Cu electroplating to fill vias without voids, and metallization on the glass wafer. In Figure 7a,b, according to the designed packaging structure, blind TGVs with a certain depth and diameter are formed economically while retaining all excellent properties. Here, we show that the picosecond laser affected zone (LAZ) has a higher etching rate while reacting with the hydrofluoric acid (HF) solution compared with the laser unaffected zone. At the LAZ, a series of nanovoids along the path of a laser beam propagating are observed, which contribute to the enhanced etchability of LAZ when the glass sample is immersed in HF solution [31]. Besides, it is easy to prepare the one-side blind TGVs by controlling the depth of focus and HF solution concentration. Here, the process parameters are set at laser beam: single-shot laser: E = μJ55 /pulse, $\tau_{p}$ = 16 ps, concentration of HF solution: 10%.

Figure 7c,d shows the cross-section view of the glass capping wafer (SCHOTT BOROFLOAT^®^33) after the LIDE. In this stage, the glass wafer with a thickness of 600 μm is thinned down to 550 μm. The feasibility of via array with 45 μm in diameter and 145 μm in depth is formed.

**Figure 8:**
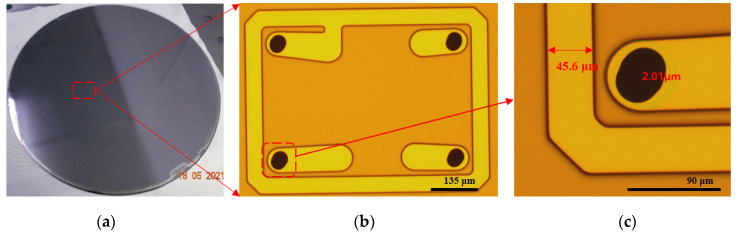
The first and second metal RDL lines. (**a**) The whole glass cap wafer with bond frame structure. (**b**) The OM image of the first RDL line. (**c**) The shift value of TGV and the first RDL line.

**Figure 9:**
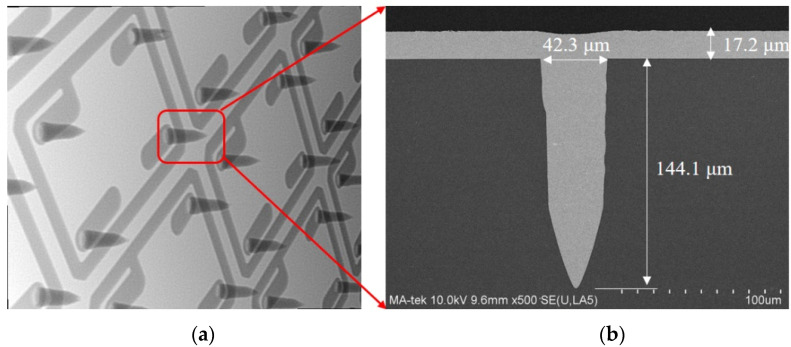
Metalized TGVs and X-ray images. (**a**) X-ray image. (**b**) Cross-sectional TGVs fully filled with Cu using bottom to up filling behavior with appropriate additive composition.

Glass is an insulating material and does not require a barrier layer before plating. After the TGVs are formed, the titanium (Ti) and Cu seed layer are sputtered on the wafer surface, including via sidewall. A negative organic film is affixed to the glass wafer and patterned. In Figure 8, the patterned photographic film was used to define the seal ring opening with a width of 45.6 μm. Meanwhile, it has high alignment accuracy (shift < 7 μm). Then the vias are deposited with Cu by full filling plating. After last, the seed layer at the non-exposed area is etched away. In Figure 9, the dimensions of TGV are 144.1 μm depth (spec value: 150 ± 10 μm), 45 μm in diameter at the via opening (spec value: 45 ± 5 μm). The aspect ratio of the via (depth-to-width of via) is ∼3:1. We have tried several combinations of plating parameters, including plating current, time, additives consisting of accelerator (A), suppressor (S), and leveler (L), to develop the void-free full-via plating. In this paper, Figure 9b shows the scanning electron microscopic (SEM) image of void-free Cu TGV plating. Large thermomechanical stresses develop at the copper-glass interface because of the significant coefficient of thermal expansion (CTE) mismatch between the copper and glass, which can lead to various reliability issues at elevated temperatures. We control the thermal mismatch between glass and copper interaction by setting proper annealing temperature with a reasonable duration. The copper via has more significant creep at higher temperatures. Therefore, a relatively higher temperature (ramp rate: 4 °C/min, peak temperature: 320 °C) is preferred in the TGV annealing process to improve the material properties of the coppe.

**Figure 10:**
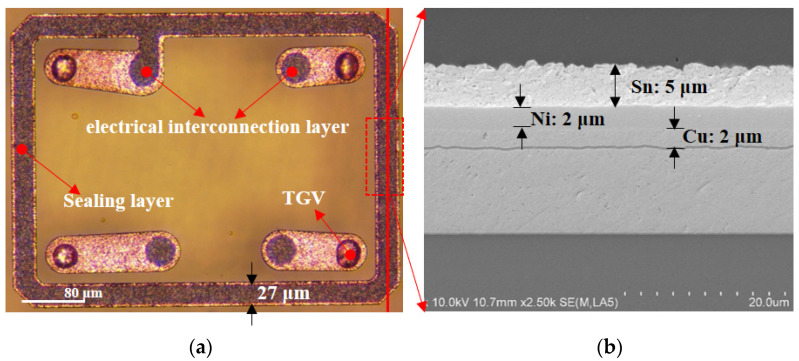
(**a**) The Cu/Ni/Sn (2/2/5) bonding layer is patterned. (**b**) The SEM micrography of a cross-section image of Cu/Ni/Sn bonding layer, which shows the thickness of plating Sn is 5 μm.

After the first RDL layer formation, the bonding layer was fabricated by photolithography and electroplating. This not only served as a sealing layer for preventing humidity or any corrosion but also acted as an electrical interconnection layer between chip pad and backside solder ball. Figure 10 presents the outlook of the chip after the bonding layer formation. The opening and thickness of the sealing layer are 27 μm and 9 μm (Cu/Ni/Sn (2/2/5 μm)), respectively.

### 3.3. Bonding Procedure

Wafer-level bonding is achieved with optimized Au-Sn TLP bonding technology with a void-free seal ring. The functional area of the RF filter, the AlN electrode, is protected in a hermetic cavity from humidity or any kind of corrosion. In the glass cap wafer, RDL and TGV vertical interconnects are processed to realize the electrical interconnection from the chip’s pad to the solder.

**Figure 11:**
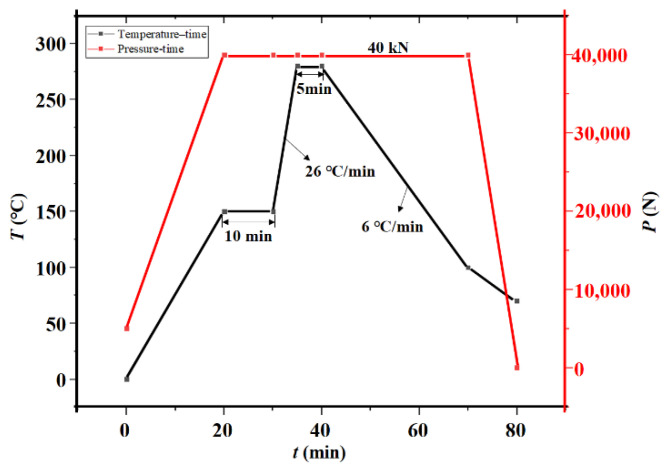
Temperature, pressure-time profile for Au-Sn bonding in the experiment.

**Figure 12:**
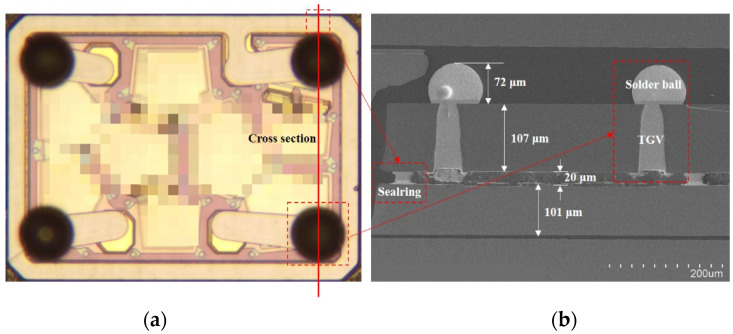
The RF filter package with TGVs after solder ball formation. (**a**) The OM picture of the RF filter package. (**b**) The cross-section view of the RF filter WLP with the solder ball.

Prior to bonding, the surface of Sn is easily oxidized in the open air at room temperature, an important step for a good bonding procedure to remove the oxide layer on the Sn surface. Surface treatments by soaking the wafer in the dilute sulfuric acid followed by a forming gas purge (typically 5%H_2_: 95%N_2_, by volume) in the bonding chamber at 100~200 °C are typically applied. There are many advantages, such as low or no outgassing from the solder layer, and it can withstand high temperature after bonding using Au-Sn bonding technology. That is, generally, the reaction between low melting temperature 232 °C Sn solder and Au will completely convert to IMC, including AuSn (δ phase) and Au5Sn (*ς*′ phase). In the package structure of this paper, the bonding structure is a 27 μm wide, 704 μm × 529 μm closed square ring with four round bumps. The design bonding interface metallization and bonding parameters are shown in Figure 11. The bonding is performed in EVG 520 vacuum bonder under a vacuum pressure around 3 × 10^−5^ mbar at 280 °C with 40 kN bonding force (approximately 6.5 MPa) for 5 min, making it advantageous for temperature-sensitive devices. Consequently, the pressure inside the sealed cavity is estimated at 3 × 10^−5^ mbar (far less than atmospheric pressure). Figure 12 shows the OM and cross-section SEM image of the final RF filter WLP. The solder ball is dropped on the opening of the UBM layer by stencil printing and reflow process. The stencil printed solder bump dimension is 100 μm, and the material is SAC320. Finally, the diameter and height of the solder ball are 100 μm and 70 μm, respectively. The wafer is diced into individual packages after the final solder balling procedure, as shown in Figure 12a. Figure 12b displays the bonding structure. No void is found in the closed square ring.

## 4. Reliability Test

### 4.1. Shear Strength Test

**Figure 13:**
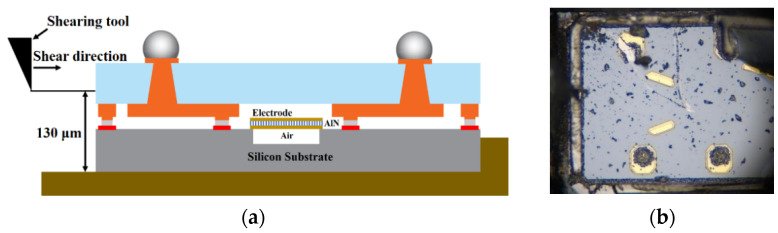
The scheme and fracture section of the die shear test scheme. (**a**) Shear strength measuring scheme; (**b**) The view of fracture after the die shear test.

**Figure 14:**
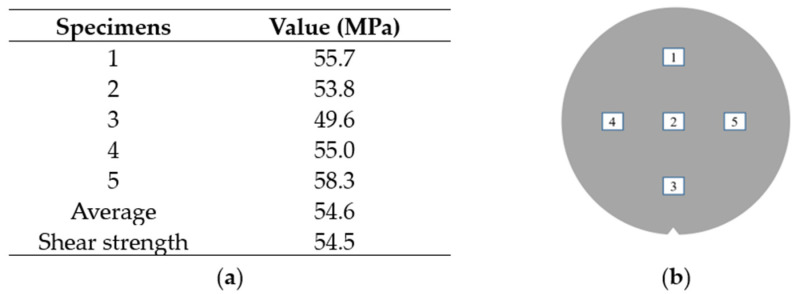
Shear strength test for RF filter WLP. (**a**) Shear force; (**b**) Measurement location.

The bonding strength of the Au-Sn joint is measured using die shear testing equipment. The test scheme is made and illustrated in Figure 13a. The bottom silicon substrate is fixed to the holder, and the shearing tool is applied to the glass cap for testing shear strength. The shear direction of the applied force is perpendicular to the glass cap die. The fracture picture after the die shear test is shown in Figure 13b. In Figure 14, the shear strength of Au-Sn bonding is measured with an average of 54.5 MPa. According to the test method standard: MIL-STD-883 (~6 MPa, method No. 2019.5Die shear strength) [29,30,31], this shear force is higher than the standard requirement. Meanwhile, J. Peng et al. [32] suggests that the mechanical reliability of Au-Sn joints during TLP bonding remains stable at 50 MPa at room temperature. In this paper, the shear strength of the TLP-bonded joint is stable, with a value of 54.5 MPa.

### 4.2. Deflection Assessment

The sealed cavity collapse of a micro-package often becomes the determining factor of the performance of encapsulated RF filters. Sealed cavity pressure comes from the molding process (appearing in the RF front-end module package), which means the maximum pressure is 3~5 MPa [33]. To predict the cavity deflection under pressure, we introduce a deflection reference model to simulate the collapse of the package capping layer [22,34].

(3) $$\omega_{\max}=\frac{5P_{\mathrm{total}}∆x^{4}∆y^{4}}{384D(∆x^{4}+∆y^{4})}$$

where $\omega_{\max}$ is the maximum deflection of the glass capping layer; ∆y and ∆x are the length and width of the cavity (475 μm × 650 μm), respectively; $P_{\mathrm{total}}$ is the total pressure on the glass capping layer; *D* is the flexural rigidity of the glass capping layer

(4) $$D=\frac{Et^{3}}{12\left(1-v^{2}\right)}$$

where *t* is the thickness of the thin glass capping layer (*t* is set as 100 μm); *E* is the young’s modulus; *v* is the Poisson’s ratio.

Applying Equation (3) to our package (with parameters in Table 2), when its pressure increases from 0 to 3 or 5 MPa. The change of the deflection of the capping layer can be derived: 0.264 μm or 1.399 μm, respectively. The highest permitted deflection change of our package is 19 μm. Our theoretical results show that the designed cavity size based on the glass capping bonding package can meet the requirements.

### 4.3. Reliability Assessment Results

According to the joint electron device engineering council (JEDEC) reliability test standards (specific details see ref. JESD22-A110E.01 [35]), in Table 3, we have also performed reliability tests on the RF filter packages to evaluate the integrity of the parts. Twenty chips are soldered onto a printed circuit board (PCB) and molding. The samples are first baked at 125 °C/24 H to remove the moisture and then soaked at 30 °C/60%/192 H. At last, reflow and uHAST (130 °C under 85% RH for 96 H) are conducted for reliability evaluation. The results show that no samples are failed.

**Figure 15:**
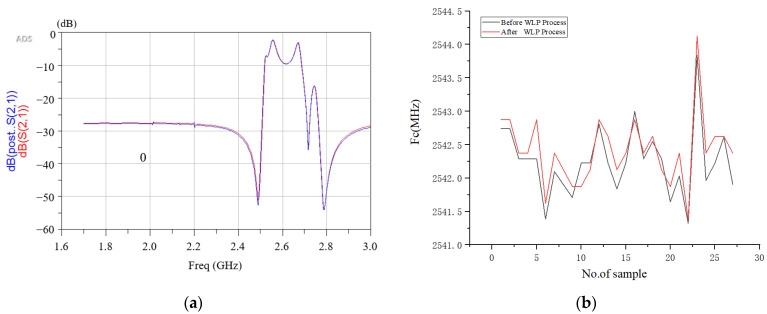
Measurement result of frequency response for RF filter (**a**) The insertion loss in passband; (**b**) The measured center frequency of RF filter.

The hermeticity of seal rings for patterned dies was evaluated by exposing them to the wafer and humidity stress test and exposure time was 96 H. The compared results before and after packaging are shown in Figure 15. We found our bonding approach for the electrical performance of the RF filter package provides the structure of the hermetic package. There is no difference in insertion attenuation across the passband (<0.2 dB, standard value: <1 dB) after the WLP process.

## 5. Conclusions

In summary, a novel RF filter wafer-level packaging solution based on Au-Sn TLP bonding technology has been proposed and realized. The proposed technology has significant advantages compared to other established processes in terms of integration of a micro-structured wafer, which can be achieved in an ultra-thin package size with a thickness of 300 μm, 1.5 μm Au bonding layer, bonding frame width as narrow as 27 μm, bonding temperatures as low as 280 °C, and a bonding force of approximately 6.5 Mpa at the same time. Due to its effective packaging process, it can provide solid reliability and reduce the manufacturing cost alternative to conventional packaging for RF filter applications. 

The main conclusions are described as follows:

(1) The glass interposer capping wafer is fabricated by LIDE, Cu plated blind TGVs, and RDL process to provide protection and electrical access to the RF filter. Following, the prepared glass capping wafer is bonded to the RF device wafer by Au-Sn TLP bonding. Shear strength’s of approx. 54.5 MPa can be determined, which is higher than the standard requirement (MIL-STD-883: method No. 2019.5 Die shear strength).
(2) The glass interposer capping wafer is fabricated by LIDE, making improving process efficiency compared to the FBAR device package using silicon lid bonding technology. In addition, the glass is transparent, we can track the packaging yield in real-time during processing.
(3) Through comparing the electrical performance after standard reliability tests, there is no difference in insertion attenuation across the passband (<0.2 dB). It is proved that the RF filter WLP with TGVs connection and cavity hermetic is a very promising solution due to its high robustness.

## 6. Patents

Zuohuan Chen, Daquan Yu, Wenbiao Ruan, et al. Wafer-level packaging structure and method for RF filter. CN Patent 202111289387.3. 11 November 2021.

## Figures and Tables

**Table 1:** The atomic diffusion concentration at the bonded interface.

Element	Spectrum1		Spectrum2		Spectrum3		Spectrum4	
	Weight%	Atomic%	Weight%	Atomic%	Weight%	Atomic%	Weight%	Atomic%
Ni	2.86	4.65	Not detected	Not detected	3.61	7.12	Not detected	Not detected
Ag	6.32	5.59	2.00	1.88	Not detected	Not detected	Not detected	Not detected
Sn	34.44	27.69	50.37	43.14	50.62	49.38	35.28	37.84
Au	50.37	24.40	42.90	22.14	43.27	25.44	62.67	40.51

**Table 2:** Glass capping layer parameters.

Items	Thickness	Young Modulus	Poisson Ratio	Critical Size
Thin glass capping layer	100 μm	64 GPa	0.3	475 μm × 650 μm

**Table 3:** Results of reliability test.

Items	Conditions		Result
Pre-Con L3	Bake	125 °C/24 H	Pass
	Soak	30 °C /60%/192 H	Pass
	Reflow	260 °C (+5/−0) 3x	Pass
uHAST	130 °C/85% RH, 96 H		Pass

## Data Availability

Not applicable.
